# Supplementary material for: A Comparison of an Australian First Nations Primary Healthcare Data Specification with Potentially Preventable Hospitalisations
Source: Int J Environ Res Public Health. 2024 Sep 9;21(9):1192. doi: 10.3390/ijerph21091192 (PMC11431243; doi:10.3390/ijerph21091192)
Supplement: Supplementary file 1 [file ijerph-21-01192-s001.zip › ijerph-3110753-SI.pdf]

Supplementary Table 1. Overlap between PPH and FNPHC specifications

PPH specification			FNPHC specification		
**PPH group**	**ICD 10**	**Description**	**FNPHC category**	**ICD10**	**Description**
	I20	Angina pectoris			
	I24.0	Coronary thrombosis not resulting in myocardial infarction			
	I24.8	Other forms of acute ischaemic heart disease			
Angina	I24.9	Acute ischaemic heart disease, unspecified	Heart disease	I20-I25	Ischaemic heart diseases
	J45	Asthma		J45	Asthma
Asthma	J46	Status asthmaticus	Asthma	J46	Status asthmaticus
Bronchiectasis	J47	Bronchiectasis	Bronchiectasis	J47	Bronchiectasis
	J41	Simple and mucopurulent chronic bronchitis			
	J42	Unspecified chronic bronchitis			
	J43	Emphysema			
COPD	J44	Other chronic obstructive pulmonary disease	COPD	J41-J44	Simple and mucopurulent chronic bronchitis; Unspecified chronic bronchitis; Emphysema; Other chronic obstructive pulmonary disease
	I11.0	Hypertensive heart disease with (congestive) heart failure	Hypertension	I10-I15	Hypertensive diseases
Congestive cardiac failure	I50	Heart failure	Heart disease	I30-I52	Other forms of heart disease
	K02	Dental caries		K02	Dental caries
	K05	Gingivitis and periodontal diseases		K05	Gingivitis and periodontal diseases
Dental conditions	K08	Other disorders of teeth and supporting structures	Oral health	K08.1	Complete loss of teeth
	E10.0–E10.9	Type 1 diabetes mellitus			
	E11.0–E11.9	Type 2 diabetes mellitus			
	E13.0–E13.9	Other specified diabetes mellitus			
Diabetes complications	E14.0–E14.9	Unspecified diabetes mellitus	Diabetes	E10-E14	Diabetes mellitus

Ear, nose and throat infections	H66	Suppurative and unspecified otitis media	Ears	H60-H95	Diseases of the ear and mastoid process
Gangrene	I70.24	Atherosclerosis of arteries of extremities with gangrene	Other circulatory disease	I70-I79	Diseases of arteries, arterioles and capillaries
Hypertension	I10	Essential (primary) hypertension	Hypertension	I10-I15	Hypertensive diseases
	I11.9	Hypertensive heart disease without (congestive) heart failure			
Iron deficiency anaemia	D50.1	Sideropenic dysphagia	Anaemia	D50-D53	Nutritional anaemias
	D50.8	Other iron deficiency anaemias			
	D50.9	Iron deficiency anaemia, unspecified			
Other vaccine-preventable conditions	B16.1	Acute hepatitis B with delta-agent (coinfection) without hepatic coma	Hepatitis B	B16	Acute hepatitis B
	B16.9	Acute hepatitis B without delta-agent and without hepatic coma			
	B18.0	Chronic viral hepatitis B with delta-agent		B18.0	Chronic viral hepatitis B with delta-agent
	B18.1	Chronic viral hepatitis B without delta-agent		B18.1	Chronic viral hepatitis B without delta-agent
Pneumonia (not vaccine-preventable)	J15.3	Pneumonia due to streptococcus, group B	Pneumonia	J12-J18	Pneumonia
	J15.4	Pneumonia due to other streptococci			
	J15.7	Pneumonia due to Mycoplasma pneumoniae			
	J16.0	Chlamydial pneumonia			
Pneumonia and influenza (vaccine-preventable)	J13	Pneumonia due to Streptococcus pneumoniae	Pneumonia	J12-J18	Pneumonia
	J14	Pneumonia due to Haemophilus influenzae			
	J10	Influenza due to other identified influenza virus	Influenza	J09-J11	Influenza
	J11	Influenza, virus not identified			
Rheumatic heart diseases	I00	Rheumatic fever without mention of heart involvement	ARF	I00-I02	Acute rheumatic fever
	I01	Rheumatic fever with heart involvement			
	I02	Rheumatic chorea			
	I05	Rheumatic mitral valve diseases	RHD	I05-I09	Chronic rheumatic heart diseases
	I06	Rheumatic aortic valve diseases			

I07	Rheumatic tricuspid valve diseases
I08	Multiple valve diseases
I09	Other rheumatic heart diseases

Supplementary Table 2. Aged-standardised rates and rate ratios for Potentially Preventable Hospitalisations.

		Age-standardised rate (per 1000)			Rate ratio (FNPHC:PPH)	
**State / Territory**	**FY**	**PPH**	**FNPHC (PDx)**	**FNPHC (Adx)**	**PDx**	**ADx**
NSW	2016-17	51.12	108.49	385.72	2.12	7.54
NSW	2017-18	53.90	110.58	377.48	2.05	7.00
NSW	2018-19	53.11	112.63	390.23	2.12	7.35
NSW	2019-20	51.02	109.46	356.60	2.15	6.99
VIC	2016-17	51.02	108.53	392.88	2.13	7.70
VIC	2017-18	59.36	114.92	386.70	1.94	6.51
VIC	2018-19	61.49	130.65	430.44	2.12	7.00
VIC	2019-20	64.66	128.09	427.04	1.98	6.60
QLD	2016-17	72.50	130.37	653.88	1.80	9.02
QLD	2017-18	78.83	134.76	576.58	1.71	7.31
QLD	2018-19	80.26	142.00	600.43	1.77	7.48
QLD	2019-20	78.41	145.98	537.56	1.86	6.86
SA	2016-17	76.23	141.65	544.87	1.86	7.15
SA	2017-18	78.47	139.22	547.88	1.77	6.98
SA	2018-19	75.42	147.37	573.96	1.95	7.61
SA	2019-20	73.15	149.50	560.16	2.04	7.66
WA	2016-17	95.65	146.19	802.90	1.53	8.39
WA	2017-18	93.50	144.21	1007.49	1.54	10.78
WA	2018-19	97.55	153.52	1177.50	1.57	12.07
WA	2019-20	93.93	146.73	1029.49	1.56	10.96
TAS	2016-17	25.04	66.56	208.49	2.66	8.33
TAS	2017-18	32.01	69.77	224.34	2.18	7.01
TAS	2018-19	29.66	74.52	225.52	2.51	7.60
TAS	2019-20	27.76	66.41	199.36	2.39	7.18
NT	2016-17	135.53	169.85	884.07	1.25	6.52
NT	2017-18	136.82	169.90	884.93	1.24	6.47
NT	2018-19	144.14	184.77	856.10	1.28	5.94
NT	2019-20	133.11	180.07	857.77	1.35	6.44
ACT	2016-17	42.82	103.14	385.72	2.41	9.01
ACT	2017-18	37.11	87.86	314.28	2.37	8.47
ACT	2018-19	37.76	102.88	368.40	2.72	9.76
ACT	2019-20	47.66	110.39	364.63	2.32	7.65
Australia	2016-17	70.91	125.52	561.08	1.77	7.91
Australia	2017-18	74.61	127.90	563.97	1.71	7.56
Australia	2018-19	75.62	134.68	597.30	1.78	7.90
Australia	2019-20	73.22	133.49	549.81	1.82	7.51

Supplementary Table 3. Age-standardised rates for all categories of FNPHC.

		Age-standardised hospitalisation rate (per 1000 persons)							
		FY 2016-17		FY 2017-18		FY 2018-19		FY 2019-20	
**FNPHC category**	**State / Territory**	**PDx**	**ADx**	**PDx**	**ADx**	**PDx**	**ADx**	**PDx**	**ADx**
Diabetes	NSW	4.36	80.24	4.71	79.51	5.15	83.27	4.96	76.76
	VIC	5.98	73.57	8.10	76.25	8.48	83.61	7.50	82.66
	QLD	7.28	143.68	6.93	151.66	8.17	159.49	8.23	133.12
	SA	6.49	123.56	7.21	125.72	8.04	134.25	7.38	123.74
	WA	8.63	259.12	8.07	361.82	9.14	440.39	9.80	382.92
	TAS	2.45	43.93	6.14	46.59	3.83	45.55	3.32	44.51
	NT	7.48	226.76	7.03	227.39	8.22	227.12	9.23	236.87
	ACT	4.92	72.70	6.63	64.76	2.27	75.09	5.84	75.08
	Australia	6.15	133.66	6.48	148.69	7.09	162.72	7.13	146.91
Respiratory health	NSW	19.98	15.48	21.25	17.41	21.93	16.67	20.83	16.15
	VIC	19.53	15.57	21.02	16.34	22.34	17.60	20.87	17.03
	QLD	24.93	18.41	26.59	20.94	27.13	20.83	25.55	21.11
	SA	31.42	20.52	32.65	23.82	32.49	28.09	30.07	24.47
	WA	35.72	25.16	34.15	22.86	37.79	27.18	32.04	22.68
	TAS	8.80	9.81	10.01	11.13	10.23	11.02	9.11	8.12
	NT	48.12	42.19	51.88	41.83	57.62	44.89	50.06	40.89
	ACT	13.11	10.60	8.15	15.69	9.59	11.80	12.36	11.41
	Australia	26.03	20.02	27.31	21.39	28.74	22.15	26.38	20.80
Circulatory health	NSW	25.46	54.54	24.15	54.62	25.25	58.10	24.68	48.15
	VIC	26.04	43.95	26.56	43.60	28.37	48.26	29.54	50.65
	QLD	33.13	80.39	34.30	87.35	34.10	86.99	34.64	70.59
	SA	25.22	60.33	27.07	64.34	30.29	67.32	28.38	58.02
	WA	37.01	92.67	34.94	89.70	37.04	89.76	34.31	72.12
	TAS	15.79	37.24	15.38	37.96	15.59	36.72	14.05	27.99
	NT	44.79	120.64	39.65	120.22	41.50	112.12	40.93	97.40
	ACT	28.05	57.98	20.25	30.57	40.15	44.82	38.33	43.79
	Australia	30.54	71.42	29.81	73.04	30.94	73.89	30.47	61.88
ARF/RHD	NSW	0.19	0.58	0.30	0.43	0.31	0.53	0.15	0.50
	VIC	0.18	0.33	0.05	0.20	0.08	0.35	0.27	0.44
	QLD	0.70	1.83	0.53	1.64	0.56	1.79	0.49	1.63
	SA	0.45	1.05	0.55	1.60	0.24	1.01	0.51	0.91
	WA	1.12	1.61	0.86	1.79	1.11	1.81	1.12	1.31
	TAS	0.27	0.29	0.07	0.12	0.04	0.15	0.14	0.39
	NT	3.86	8.18	4.17	7.81	4.55	7.36	3.58	5.92
	ACT	0.00	0.00	0.00	0.17	0.00	0.51	0.00	0.00

FNPHC category	State / Territory	Age-standardised hospitalisation rate (per 1000 persons)							
		FY 2016-17		FY 2017-18		FY 2018-19		FY 2019-20	
		PDx	ADx	PDx	ADx	PDx	ADx	PDx	ADx
	Australia	0.82	1.79	0.81	1.68	0.84	1.69	0.72	1.44
CKD	NSW	1.78	13.24	1.57	14.40	0.71	14.97	1.30	11.51
	VIC	0.75	28.32	0.60	18.90	0.78	21.78	1.02	23.72
	QLD	1.22	146.35	1.03	36.80	1.32	36.85	1.52	23.99
	SA	2.19	28.58	3.08	32.35	2.26	35.82	2.54	40.08
	WA	2.37	100.52	2.58	205.85	2.34	285.91	2.31	213.65
	TAS	0.34	4.86	0.19	4.83	0.02	5.24	0.42	5.32
	NT	3.27	83.20	4.51	86.83	6.66	88.85	6.71	86.67
	ACT	0.82	33.19	0.94	10.27	1.06	17.83	0.18	9.90
	Australia	1.73	67.56	1.80	51.78	1.73	62.49	2.01	49.05
Eyes & ears	NSW	12.45	5.89	12.91	5.70	13.73	6.57	12.30	5.47
	VIC	10.70	5.54	12.31	5.93	13.69	6.06	10.35	5.24
	QLD	15.55	8.98	15.18	9.59	17.09	9.92	17.25	9.39
	SA	12.35	8.96	13.06	9.53	15.40	10.50	11.91	8.14
	WA	16.16	11.71	17.93	10.88	16.66	10.21	16.22	10.48
	TAS	10.15	4.55	8.22	7.30	10.95	4.63	6.18	3.35
	NT	18.26	21.23	17.14	17.81	18.47	16.69	15.22	15.33
	ACT	9.88	6.22	11.84	5.51	9.62	6.08	4.78	5.25
	Australia	14.08	8.95	14.32	8.80	15.42	8.97	13.95	8.09
Oral health	NSW	1.64	0.66	1.81	0.75	1.82	0.85	1.59	0.76
	VIC	2.24	0.68	2.87	0.97	2.74	0.77	2.44	0.66
	QLD	1.72	1.26	1.86	1.20	2.13	1.38	1.81	1.32
	SA	2.77	0.44	2.23	1.34	2.65	0.89	2.58	1.31
	WA	1.40	2.77	0.99	2.55	1.09	2.87	1.36	2.73
	TAS	1.53	0.48	1.81	0.52	1.79	0.72	1.94	0.55
	NT	2.53	3.30	2.43	2.65	2.84	2.61	2.55	2.31
	ACT	2.16	1.49	2.83	1.61	3.74	1.01	2.49	1.15
	Australia	1.80	1.33	1.88	1.32	2.02	1.41	1.83	1.32
STI & blood-borne diseases	NSW	0.13	18.47	0.14	16.78	0.20	15.36	0.17	13.98
	VIC	0.32	25.30	0.30	23.24	0.27	25.51	0.30	23.72
	QLD	0.31	19.17	0.33	21.77	0.31	20.52	0.35	16.88
	SA	0.36	34.62	0.29	26.41	0.29	26.76	0.29	23.96
	WA	0.38	25.21	0.43	25.88	0.52	26.93	0.45	29.74
	TAS	0.00	3.57	0.03	5.51	0.08	4.69	0.04	5.49
	NT	1.04	32.78	0.71	31.93	0.92	28.17	0.63	26.99
	ACT	0.00	22.37	0.40	22.73	0.09	18.24	0.00	18.22
	Australia	0.32	21.74	0.30	21.57	0.35	20.51	0.31	19.25
Cancer	NSW	3.86	4.14	4.17	4.57	4.25	4.61	4.31	5.87

FNPHC category	State / Territory	Age-standardised hospitalisation rate (per 1000 persons)							
		FY 2016-17		FY 2017-18		FY 2018-19		FY 2019-20	
		PDx	ADx	PDx	ADx	PDx	ADx	PDx	ADx
	VIC	4.46	13.49	3.97	19.75	5.45	23.89	5.74	17.68
	QLD	4.64	16.66	4.69	17.45	4.59	21.02	5.08	21.77
	SA	5.80	8.20	3.91	9.51	5.86	7.99	4.88	7.58
	WA	2.87	12.09	3.23	10.28	3.87	10.96	3.28	11.88
	TAS	3.66	4.57	3.45	5.74	3.84	6.28	4.55	6.10
	NT	3.47	12.70	2.74	9.67	3.29	10.88	3.03	12.46
	ACT	4.80	8.83	4.35	1.65	4.48	1.04	4.37	2.34
	Australia	4.08	10.09	4.04	10.52	4.38	11.94	4.42	12.38
Depression & suicide	NSW	3.09	7.85	3.29	6.75	4.05	6.43	3.22	5.78
	VIC	3.24	6.82	3.24	6.68	4.97	7.96	4.70	7.16
	QLD	4.03	7.57	3.68	8.00	3.48	8.31	4.71	8.57
	SA	4.67	10.20	4.33	10.45	3.75	9.98	4.04	8.97
	WA	2.98	5.57	2.40	5.79	2.95	6.45	2.65	5.75
	TAS	5.22	6.00	6.34	5.06	6.26	3.36	6.40	3.23
	NT	0.93	5.74	1.25	5.06	1.10	6.56	0.98	5.79
	ACT	3.94	8.46	1.88	9.22	1.52	6.98	3.39	7.57
	Australia	3.31	7.30	3.26	7.03	3.59	7.21	3.63	6.81
Mental disorders	NSW	15.47	19.85	16.49	19.76	15.95	21.20	16.09	18.93
	VIC	16.19	20.02	16.02	17.17	19.16	22.71	19.89	23.69
	QLD	14.31	18.09	15.87	21.86	17.26	24.42	20.39	24.31
	SA	22.66	20.54	19.36	22.13	19.42	23.96	24.34	22.80
	WA	13.56	14.00	13.95	14.48	14.23	15.77	16.04	16.14
	TAS	7.66	12.98	7.67	13.26	11.82	15.13	9.58	12.18
	NT	9.51	12.52	10.56	15.52	12.12	16.57	14.57	18.03
	ACT	14.27	17.94	12.28	14.96	12.55	19.82	17.94	19.31
	Australia	14.59	17.76	15.31	18.97	16.09	21.01	17.80	20.31
Mental-health related	NSW	0.66	8.46	0.69	8.52	0.87	9.25	0.65	6.94
	VIC	0.81	8.48	1.20	6.57	1.00	8.07	1.02	7.73
	QLD	0.66	7.77	0.88	9.71	0.97	12.12	0.87	11.67
	SA	0.58	8.83	0.97	9.12	0.75	10.14	1.18	9.83
	WA	0.79	7.48	0.68	7.69	0.63	8.69	0.51	8.35
	TAS	0.33	3.28	0.21	2.73	0.35	3.99	0.08	2.47
	NT	0.86	7.48	0.85	8.11	1.09	8.37	1.17	8.00
	ACT	1.37	6.43	0.24	6.08	0.45	7.32	0.43	7.65
	Australia	0.71	7.92	0.80	8.40	0.87	9.65	0.79	8.61
Alzheimer & dementia	NSW	0.40	2.24	0.35	1.89	0.40	2.29	0.44	2.18
	VIC	0.09	1.16	0.37	1.53	0.37	1.54	0.42	1.89
	QLD	0.99	5.90	0.38	4.98	0.69	4.97	0.55	4.74

FNPHC category	State / Territory	Age-standardised hospitalisation rate (per 1000 persons)							
		FY 2016-17		FY 2017-18		FY 2018-19		FY 2019-20	
		PDx	ADx	PDx	ADx	PDx	ADx	PDx	ADx
	SA	0.50	2.08	0.88	2.88	0.48	2.52	0.69	2.73
	WA	0.63	4.41	0.84	3.46	0.70	4.42	0.64	4.51
	TAS	0.12	0.70	0.12	0.99	0.11	1.07	0.36	1.52
	NT	0.60	5.85	0.69	6.89	0.47	5.15	0.79	6.38
	ACT	0.00	2.77	0.00	0.17	0.00	0.44	0.00	4.00
	Australia	0.56	3.55	0.46	3.24	0.50	3.34	0.52	3.44
Alcohol & drugs	NSW	14.86	42.29	13.66	38.12	12.62	37.70	13.74	36.27
	VIC	11.93	32.82	10.21	29.84	14.33	35.01	15.38	36.99
	QLD	14.80	50.13	15.88	53.16	16.95	56.09	17.55	55.29
	SA	22.88	74.24	20.45	67.73	22.26	62.62	27.58	73.24
	WA	17.78	67.33	17.18	67.34	19.23	63.40	20.52	62.89
	TAS	7.37	13.76	6.76	15.91	6.28	16.75	5.20	13.58
	NT	21.70	105.24	23.24	102.02	21.99	94.70	26.28	99.36
	ACT	15.69	41.44	14.87	36.40	12.56	39.87	15.58	43.14
	Australia	16.08	54.78	15.87	53.40	16.24	52.83	17.82	53.16
Older adults (50+)	NSW	0.06	3.34	0.10	2.82	0.08	3.22	0.16	3.04
	VIC	0.19	3.44	0.30	3.76	0.37	4.52	0.22	4.37
	QLD	0.15	4.22	0.18	4.37	0.20	4.57	0.23	4.99
	SA	0.00	2.98	0.00	4.82	0.00	4.06	0.06	4.49
	WA	0.03	4.61	0.21	5.05	0.11	3.82	0.11	3.71
	TAS	0.13	1.44	0.11	1.51	0.00	2.15	0.00	1.97
	NT	0.11	5.78	0.02	7.22	0.09	6.44	0.09	6.97
	ACT	0.00	3.94	0.00	1.57	0.00	1.35	0.00	4.58
	Australia	0.09	3.85	0.14	3.97	0.13	4.01	0.16	4.13
Lifestyle factors	NSW	4.10	108.47	4.99	105.45	5.30	109.24	4.87	104.33
	VIC	5.89	113.41	7.81	115.96	8.23	122.80	8.42	123.43
	QLD	5.97	123.46	6.44	126.11	7.06	131.16	6.76	128.18
	SA	3.30	139.75	3.19	136.15	3.18	148.04	3.06	149.87
	WA	4.73	168.65	5.78	172.05	6.10	178.91	5.39	180.62
	TAS	2.74	61.03	3.26	65.17	3.32	68.06	5.04	62.60
	NT	3.33	190.48	3.04	193.97	3.84	179.64	4.26	188.39
	ACT	4.11	91.34	3.21	92.93	4.80	116.17	4.71	111.25
	Australia	4.62	129.38	5.32	130.17	5.75	133.48	5.56	132.24
FNPHC total	NSW	108.49	385.72	110.58	377.48	112.63	390.23	109.46	356.60
	VIC	108.53	392.88	114.92	386.70	130.65	430.44	128.09	427.04
	QLD	130.37	653.88	134.76	576.58	142.00	600.43	145.98	537.56
	SA	141.65	544.87	139.22	547.88	147.37	573.96	149.50	560.16
	WA	146.19	802.90	144.21	1007.49	153.52	1177.50	146.73	1029.49

FNPHC category	State / Territory	Age-standardised hospitalisation rate (per 1000 persons)							
		FY 2016-17		FY 2017-18		FY 2018-19		FY 2019-20	
		PDx	ADx	PDx	ADx	PDx	ADx	PDx	ADx
	TAS	66.56	208.49	69.77	224.34	74.52	225.52	66.41	199.36
	NT	169.85	884.07	169.90	884.93	184.77	856.10	180.07	857.77
	ACT	103.14	385.72	87.86	314.28	102.88	368.40	110.39	364.63
	Australia	125.52	561.08	127.90	563.97	134.68	597.30	133.49	549.81
